# CD44 as a tumor biomarker and therapeutic target

**DOI:** 10.1186/s40164-020-00192-0

**Published:** 2020-12-10

**Authors:** Hanxiao Xu, Mengke Niu, Xun Yuan, Kongming Wu, Aiguo Liu

**Affiliations:** 1 Department of Pediatrics, Tongji Hospital of Tongji Medical College, Huazhong University of Science and Technology, Wuhan, 430030 China; 2 Department of Medical Oncology, The Affiliated Tumor Hospital of Zhengzhou University: Henan Cancer Hospital, Zhengzhou, 450008 China; 3 Department of Oncology, Tongji Hospital of Tongji Medical College, Huazhong University of Science and Technology, 1095 Jiefang Avenue, Wuhan, 430030 People’s Republic of China

**Keywords:** CD44, Cancer, Cancer stem cells, Epithelial-mesenchymal transition, Tumor initiation, Cancer progression, Therapy-resistance, Targeted therapy

## Abstract

CD44, a complex transmembrane glycoprotein, exists in multiple molecular forms, including the standard isoform CD44s and CD44 variant isoforms. CD44 participates in multiple physiological processes, and aberrant expression and dysregulation of CD44 contribute to tumor initiation and progression. CD44 represents a common biomarker of cancer stem cells, and promotes epithelial-mesenchymal transition. CD44 is involved in the regulation of diverse vital signaling pathways that modulate cancer proliferation, invasion, metastasis and therapy-resistance, and it is also modulated by a variety of molecules in cancer cells. In addition, CD44 can serve as an adverse prognostic marker among cancer population. The pleiotropic roles of CD44 in carcinoma potentially offering new molecular target for therapeutic intervention. Preclinical and clinical trials for evaluating the pharmacokinetics, efficacy and drug-related toxicity of CD44 monoclonal antibody have been carried out among tumors with CD44 expression. In this review, we focus on current data relevant to CD44, and outline CD44 structure, the regulation of CD44, functional properties of CD44 in carcinogenesis and cancer progression as well as the potential CD44-targeting therapy for cancer management.

## Background

Over the past decades, the conception that carcinoma represents a malignant disease type with both phenotypic and genetic heterogeneity has become completely accepted in the field of oncology. Although tremendous advancement has been achieved in precise management of this progressive disease [1–3], local invasion, distant metastasis and therapy resistance hinder survival improvement among tumor patients. A flurry of research has sprung up in order to throw light upon the underlying molecular mechanisms.

Cluster of differentiation 44 (CD44) is a complex transmembrane adhesion glycoprotein, and fundamentally associates with the pivotal component of the extracellular matrix (ECM) hyaluronic acid (HA) [4]. CD44 expresses in a variety of cell types in humans, including embryonic stem cells, differentiated cells and cancer cells [5]. Distinct alternative splicing during the transcription process produces two isoforms of CD44, including the standard isoform (CD44s) and CD44 variant isoforms (CD44v) [6].

Numerous studies have reported that CD44 not only prominently participates in normal cellular functions during physiological processes [7, 8], but also plays pivotal roles in pathological processes, especially tumors [9]. CD44 plays important roles in diverse physiological processes, such as organ development, diverse immune functions and haematopoiesis [10]. CD44-mediating processes include T cell differentiation, branching morphogenesis, proliferation, adhesion and migration [10]. For instance, loss-of-function of CD44 in mice contributed to abnormalities in bone-marrow colonization [11] as well as in the migration of lymphocytes to lymph nodes or the thymus [12]. As observed in pregnant mice, CD44-deletion impaired the preservation of lactation post-partum and accelerated uterine involution [13]. In another study, CD44 depletion suppressed the proliferation of smooth muscle cells in mice as comparison to wild-type controls [14].

It has been evident that CD44 as a surface biomarker of cancer stem cells (CSCs) and a vital regulatory factor of epithelial-mesenchymal transition (EMT) program is involved in the regulation of tumor initiation and development [6, 15–17]. Aberrant expression of CD44 and dysregulation of CD44 contribute to tumor formation of multiple cancer entities, including lung cancer [18], hepatocellular carcinoma [19], ovarian cancer [20], glioma [21], papillary thyroid carcinoma [22], head and neck squamous cell carcinoma (HNSCC) [23], astrocytic gliomas [24] and oral squamous cell carcinoma (OSCC) [25]. In hepatocellular carcinoma cells (HuH7) which originally express CD44s rather than CD44v, silence of CD44 gene impaired the potential of spheroid formation and enhanced sensitivity to sorafenib and 5-fluorouracil (5‐FU), accompanied by remarkable downregulation of CSC-related genes including CD133 and EpCAM [19]. The 3′ untranslated region of CD44, acting as a competing endogenous RNA to microRNA-34a, boosts the sensitivity of liver CSCs to natural kill cells-mediated cytotoxicity via regulating UL16 binding protein 2 [26]. In addition, CD44 also exerts significant effects on caner invasion and metastasis of various tumor types [27], such as lung adenocarcinoma [18], breast cancer [28–30], neuroblastoma [31], gastric cancer [32], esophageal squamous cell carcinoma (ESCC) [33], colorectal cancer [34–37], prostate cancer [38], nasopharyngeal carcinoma [39], endometrial cancer [40], clear cell renal cell (RCC) carcinoma [41], pancreatic cancer [42], meningioma [43] and ovarian cancer [44]. As has been reported, the intracellular domain (ICD) of CD44 interacting with RUNX2 to form a co-transcription factor drives the migration of prostate cancer cell line PC3 through upregulating the levels of metastasis-related genes, such as matrix metalloproteinase 9 (MMP9) and osteopontin [38]. However, CD44 epithelial isoform has been found to be negatively correlated with lymphatic invasion and metastasis of colorectal cancer based on the statistical analysis of a total of 494 colorectal tumor samples [34]. Besides, numerous studies suggest that CD44 can be a promising predictor for clinical outcomes among cancer population, including gastric cancer [45, 46], colorectal cancer [34], neuroblastoma [31], myxofibrosarcoma [47], glioma [21], endometrial cancer [40] and osteosarcoma [48]. According to survival analysis, grade II/III glioma patients with high mRNA expression of CD44 experienced poor overall survival (OS) and progression-free survival (PFS) in comparison with low mRNA level of CD44 in an independent manner [21]. Also, CD44 has been found to be correlated with unfavorable PFS and cancer-specific survival in non-muscle-invasive papillary upper tract urothelial carcinoma [49].

This review aims to encapsulate the structure of CD44 gene and protein, outline the roles which CD44 plays in tumor initiation, progression and therapy-resistance, and also highlight the perspectives for CD44-targeted therapy.

## The structure and function of CD44

CD44, which is encoded by CD44 gene on the short arm of chromosome 11 in human, is a type of complex cell-surface glycoprotein [6]. The CD44 gene is composed of 19 exons in human, among which the first five exons (exons 1–5) and the last five exons (exons 16–20) constantly encode CD44s which is the most common and the smallest CD44 protein with a molecule weight of 85–95 kDa [50]. The exons 1–5 and exons 16–20, regarded as stable exons, encode N-terminal containing HA-binding region and C-terminal domain of CD44 protein, respectively [51]. The middle nine exons can be alternatively spliced and located between exons 1–5 domain and exons 16–20 region, which form multiple different permutations and subsequently encodes manifold CD44v.

**Fig. 1:**
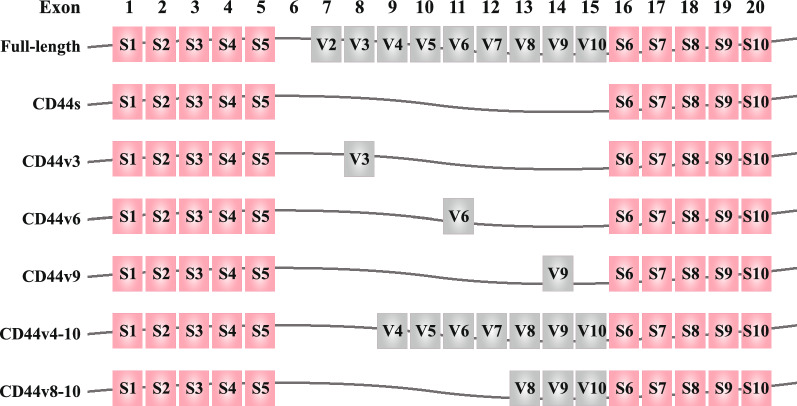
Structure of CD44 gene. Human CD44 is encoded by 19 exons with the absence of exon 6. Pink color exons constantly encode CD44s. The middle gray color exons can be inserted by alternative splicing and form diverse CD44v. Full-length CD44, CD44s, CD44v3, CD44v6, CD44v9, CD44v4-10 and CD44v8-10 are displayed schematically

**Fig. 2:**
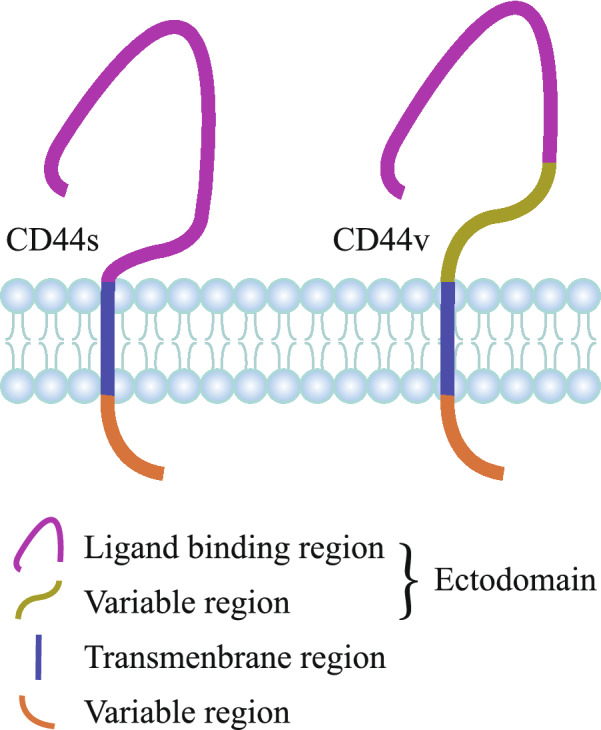
Structure of CD44 protein. CD44 mainly consists of three regions, including extracellular domain, transmembrane domain and intracellular domain. Compared to CD44s, the extracellular region of CD44v protein additionally contains a variable domain

CD44 consists of three regions, including ectodomain, transmembrane region and intracellular tail [5, 52]. CD44v isoforms contain an additional stem membrane-proximal portion which is encoded by a single variant exon or multifarious conceivable combinations of variant exons. The CD44 peptide can be further processed by glycosylation and addition of heparin sulfate or chondroitin sulfate [53, 54]. The structure schematic diagrams of CD44 gene and CD44 protein are shown in Figs. 1, 2, respectively.

Although CD44 itself is inherently lack in kinase activity, it can participate in signaling via specifically interacting with kinases and other signaling molecules [4]. Contrary to common acknowledgements that the intact CD44 translocates to the cell nucleus and then regulates transcription, a recent research has shown that CD44 is firstly cleaved and then the cytoplasmic domain rather than the intact CD44 translocates to the nucleus [4].

CD44 is a well-known marker of CSCs and plays important roles in tumor initiation and development [55]. CD44 has been implicated in driving CSC traits through activating platelet-derived growth factor receptor β/signal transducer and activator of transcription 3 (STAT3) signaling [56]. CD44s, as the primary isoform in breast CSCs, is correlated with gene profile of CSCs [56]. Knockdown of CD44s impairs the properties of CSCs, and the alternative splicing from CD44v to CD44s induces the genotype and phenotype of CSCs [56]. Emerging evidence has demonstrated that CD44 can serve as a stimulator or promoter for EMT process through regulating related pathways [6]. As has been observed, mesenchymal breast CSCs featured with CD24(−)/CD44( +) localize at the invasive front of tumors, while epithelial-like breast CSCs with aldehyde dehydrogenase 1 are more located in the center of tumors [57].

## The regulatory network referring to CD44

### CD44 ligands

There are several common ligands to which CD44 binds, including HA, fibronectin [58], serglycin/sulfated proteoglycan [59], osteopontin [60] and chondroitin [61]. HA, a primary ECM constituent, represents the most common ligand of CD44 which contains a HA-binding site in the N-terminal region of extracellular domain. The combination of HA and CD44 facilitates binding of adaptor molecules to cytoplasmic region in CD44, and activates multiple pathways involved in cell adhesion, migration and proliferation [10], including Ras, mitogen-activated protein kinases (MAPK) and phosphoinositide 3-kinase (PI3K) [62]. As has been reported, HA binding to CD44 enhances proliferation and survival of breast cancer cells through modulating β-catenin signaling and nuclear factor-kappa B (NFκB)-specific transcription activity and subsequently promoting the expression of P-glycoprotein and B-cell lymphoma-extra large (Bcl-xl) genes in breast cancer MCF-7 cells [63]. Bladder cancer HT1376 cells with the transfection of HA synthase 1-anti sense displayed remarkably decreased protein and mRNA expression of CD44v3, CD44v6 and CD44s in comparison with the control counterpart, which indicated that HA synthase modulated bladder cancer growth, invasion and angiogenesis through regulating HA synthesis and the expression of its receptor CD44 [63].

Fibronectin does not bind to CD44 directly as HA does. CD44 which was firstly combined with HA interacts with fibronectin in ECM. Suppression of HA synthesis contributed to fibronectin and collagen deposition as well as regulated transforming growth factor-β1 (TGF-β1)-mediated lung myofibroblasts [64]. Among colon cancer SW480 cells, the extra domain A of fibronectin drove tumorigenesis by maintaining the properties of CD133 + /CD44 + subgroup [65].

In hematopoietic cells, serglycin has been reported to be capable of binding specifically to CD44, which depends on CD44 activation [59]. Glycosaminoglycans consisting of chondroitin sulfate are combined with serglycin and promote CD44 binding. The interaction with CD44 active form promotes the degranulation of CD44-positive cytotoxic T lymphocyte clones, and modulates lymphoid cell adherence and activation [66].

Osteopontin is a plasma protein and predicts adverse prognosis of multiple cancer types such as stomach tumor, thyroid carcinoma and breast cancer [67]. Osteopontin can also bind to CD44 and subsequently promotes cell signaling involved in tumor progression and metastasis [60]. It has been observed that osteopontin expression is correlated with CD44 level in lung small cell lung cancer [68] and gastric cancer [69]. As reported, osteopontin maintains the “stem” properties and drives radiation resistance through activating CD44 in adjacent glioma cells [70]. In addition, osteopontin also enhances the expression of its receptors CD44s and CD44v6 [71]. Interaction between osteopontin and CD44s/CD44v activates phospholipase C-γ-dependent protein kinase B (Akt) pathway, which promotes the motility and survival of cancer cells [72].

### Molecules and pathways that CD44 regulates

CD44 participates in a diversity of signaling and pathways involved in both physiological and pathological processes, especially in carcinogenesis and tumor progression. CD44s has been implicated in regulating expression of MMPs in the HA-dependent or HA-independent manner, especially MMP2 [73, 74] and MMP9 [75]. Aberrant expression of MMPs which represent a family of endopeptidases and can degrade ECM, facilitate tumor invasion and metastasis [76, 77]. The work by Miletti-González KE, et al. revealed that CD44-ICD rather than intact CD44, firstly translocated into nucleus, subsequently bound to CD44-ICD response element, and finally promoted the transcriptional expression of MMP9 [78]. CD44-ICD also drove the expression of three oxidative glycolysis-related vital genes (ALDOC, 3-Phosphoinositide-dependent kinase 1 (PDK1) and 6-phosphofructo-2-kinase/fructose-2,6-bisphosphatase 4 (PFKFB4)), maintaining the metabolic needs for cancer cell survival preferentially through oxidative glycolysis rather than citric acid cycle even in the condition of adequate oxygen supply [78]. In addition, CD44-ICD can also act as an organizer for recruiting the receptor tyrosine kinase c-Met, hepatocyte growth factor (HGF)/scatter factor (SF) and CD44 to form a ternary complex, and assemble proteins ezrin/radixin/moesin (ERM) which are essential to signaling transferring from c-Met to mitogen-activated protein kinase (MEK) and extracellular signal-regulated kinase (Erk) [79]. Furthermore, both CD44v6 and ERM can interact with vascular endothelial growth factor receptor (VEGFR) which contributed to angiogenesis [80].

CD44 has been reported to regulate the levels of cell cycle-related proteins, including cyclin D [81] and cyclin A [82]. CD44 can enhance the proliferation of gastric stem cells through upregulating cyclin D1 and then driving the progression from cell cycle stage G1 phase to S phase [46]. De Falco et al. has reported that CD44-ICD up-regulates cyclin D1 expression through promoting CREB (a transcription factor) recruitment to the cyclin D1 promoter and driving cyclin D1 transcription, and ultimately accelerates cell proliferation in thyroid carcinoma [83].

Down-regulation of CD44 reduced β-catenin expression and enhanced the level of phosphorylated β-catenin [81]. Increased expression of phosphorylated β-catenin contributed to the instability of Wnt/β-catenin pathway, subsequently decreasing nuclear accumulation in both chronic myeloid leukemia K562 cells and corresponding nude mouse transplantation model [81]. In addition, CD44 can promote the disassociation of E-cadherin (an epithelial marker) and β-catenin on plasma membrane, facilitate translocation of the released β-catenin, and then activate genes driving cell migration and invasion [84]. Furthermore, abnormal activation of the PI3K-Akt pathway was observed in CD44-positive pediatric posterior fossa ependymoma [85], suggesting CD44 might be involved in the regulation of PI3K-Akt pathway. Besides, CD44 positively modulates the expression of nuclear factor erythroid 2-like 2 (a key regulator of antioxidant genes) in doxorubicin-resistant breast cancer cell lines [86].

PD-L1 draws much attention as an immune checkpoint for improving cancer management [87]. CD44 positively regulated PD-L1 expression in triple-negative breast cancer (TNBC) and non-small cell lung cancer through activating PD-L1 transcription partly via the association between its intracytoplasmic domain and a regulatory region in PD-L1 [87].

### Molecules and pathways which modulate CD44

On the other hand, CD44 also can be regulated by various molecules, signaling and some drugs. Some microRNAs have been found to negatively modulate CD44 expression, such as microRNA-150 [39], microRNA-200c [88], microRNA-34a [33], microRNA-330-5p [89], microRNA-145 [90], microRNA-3129 [91], microRNA-143 [92], microRNA-328 [93], and microRNA-373 [94]. CD44 has been reported to be a novel downstream target of microRNA-150 in nasopharyngeal carcinoma [39]. The modulator of Wnt signaling (ICG-001) enhanced the expression of microRNA-150 which acts as a negative regulator of CD44, resulting in the suppression of cancer cell migration [39]. Another study reported that scaffold/adaptor growth factor receptor bound 2-associated binding protein 2 (GAB2)-PI3K signaling enhanced EMT characteristics and the expansion of CSC-like cells via microRNA-200c/CD44 axis in ovarian cancer [88]. Also, in human ESCC as reported, microRNA-34a decreased CD44 expression through interacting with a putative binding site of CD44 3′ untranslated region, and CD44 knockdown could reverse the facilitation effects of microRNA-34a knockdown on tumor growth and metastasis of human ESCC cells (ECA109 and TE-13) [33]. However, some other microRNAs have been recognized to positively regulate CD44 in tumors, such as microRNA-492 [95]. In liver cancer cells (HepT1 and HUH7), microRNA-492 strongly directly enhanced the expression of both CD44s and CD44v10 [95]. In human ovarian cancer tissues and cells (SKOV3), microRNA-21 enhanced the level of CD44v6 through activating Wnt signaling, resulting in the promotion of proliferation, invasion and migration [20].

CD44 is also regulated by interleukin (IL)-4. IL-4 exposure (5 ng/ml) enhanced CD44 expression among prostate cancer cells PC3 [96]. Besides, krüppel-like factor 4 negatively modulated CD44 expression via binding to the CD44 promoter and disturbing gene transcription to restrict metastatic properties in human pancreatic ductal adenocarcinoma cells [97]. Additionally, spalt like transcription factor 4 regulates CD44 alternative splicing by increasing KH RNA binding domain containing signal transduction associated 3 (KHDRBS3), a splicing factor for CD44 [98]. CD44v overexpression reversed a reduction in the sphere formation ability induced by KHDRBS3 knockdown, indicating that CD44v plays promoting roles in cancer stemness [98]. Besides, glucose-regulated protein 78 acts as an interplay partner and a regulator of CD44v membrane homeostasis, and modulates cell spread among tamoxifen-resistant breast cancer cells [99]. Treatment of ESCC cells with TGF-β which is an inductor of EMT, significantly increased CD44v9 expression [100].

CD44 has also been reported to be a direct target of nicotinamide N-methyltransferase (NNMT) in hepatocellular carcinoma [101]. Through changing the histone H3 methylation on 27 methylation pattern and activating CD44 at transcriptional level, NNMT displays significantly promotes vascular invasion and distant metastasis of hepatocellular carcinoma [101]. The underlying molecular mechanism that NNMT induces N6-methyladenosine modification of CD44 mRNA and prevents ubiquitin-induced degradation lies behind that NNMT leads to the formation of CD44v3 and stabilization of CD44 molecule, respectively [101].

Metformin, a well-known first-line drug for diabetes treatment, was found to be capable of down-regulating CSC marker CD44 in primary oral cancer cells [102]. Besides, O-glycosylation is known to play important roles in body development, cell adhesion and tumorigenesis [103]. In Gao T’s study, exosomes were isolated from human colon cancer cells (LS174T and LSC) [103]. It was found that higher level of CD44 was detected in exosomes from aberrant O-glycosylated cells in comparison with normal counterparts [103]. However, the tendency of CD44 expression inside these cells was surprisingly completely contrary to that in exosomes [103]. These results indicate that abnormal O-glycosylation inversely regulated the expression of CD44 [103].

### CD44 and tumor initiation

The phenomenon observed in a variety of cancer types that differential expression of CD44 between tumors and corresponding normal counterparts might potently indicate that CD44 plays essential roles in tumorigenesis [104].

As has been observed, the expression of CD44 was significantly lower in OSCC in comparison with oral lichen planus which is a precancerous state of OSCC, indicating that CD44 might trigger the malignant conversion from precancerous state to cancer [105]. In pleomorphic adenoma, knockdown of CD44 not only impaired the malignant behaviors of tumor-initiating cells in vitro, but also suppressed tumorigenesis in xenograft mice [106]. Among human colorectal cancer (COLO 201) cells, CD44 positive cells displayed remarkably enhanced capacity of tumor formation in compared with CD44 negative counterparts in immunodeficient mice [107]. Interestingly, although CD44 serves as a biomarker of tumor-initiating cells with high expression of CD44, overexpression of CD44 cannot endow low CD44-expressing cells with properties of tumor-initiating cells [106]. Besides, CD44^high^/CD49^high^ subpopulation of prostate cancer PC3 cells displayed enhanced proliferative and clonogenic potential as compared to the CD44^low^/CD49^low^ subgroup [96].

Debanjan Dhar’s work has thrown light upon the pivotal role of CD44 in the tumorigenesis of hepatocellular carcinoma [108]. As reported, there was a sharp rise in CD44 expression in hepatocytes relying on the presence of STAT3 when these cells were exposed to carcinogen [108]. Subsequently, CD44 activates AKT to promote MDM2 phosphorylation and its subsequent nuclear translocation, which switches off p53 genomic surveillance response [108]. Following that the genomic surveillance function of p53 was seriously interfered, damaged hepatocytes can escape from p53-mediated death and respond to proliferation-related signals, which induce maintenance of mutations and transmission of these mutations from parental cells to daughter cells [108]. These daughter cells further become hepatocellular carcinoma progenitors, and hepatocellular carcinoma occurs ultimately [108]. Another study based on a three-dimensional Matrigel model showed that melanoma cells experienced specialized cells-mediated coalescence while non-tumorigenic cells did not undergo coalescence, which provides evidence to support that coalescence was recognized to be a peculiarity of tumorigenic cells. Blocking CD44 with a specific antibody hamper tumorigenic cells to coalesce, which indicates another mechanism underlying the promoting roles of CD44 in cancer initiation [109].

### CD44 and tumor development

CD44 plays essential roles in cancer progression of multiple tumor types, including breast cancer [110], lung adenocarcinoma [18], ovarian cancer [20], and glioblastoma [111]. Invasion from in situ to adjacent tissues of tumor cells occurs before metastasis and contributes to cancer development [110]. It has been found that CD44^high^ cancer cells are among the chief subgroup of collectively invading luminal breast tumor cells with distinctive gene profile of mesenchymal genes and pivotal functional regulators of invasion [110]. Intriguingly, the conversion from CD44^low^ to CD44^high^ was along with a shift from CD44s to CD44v, rather than a shift from non-CSCs to CSCs [110]. It has been recognized that CTC clusters grew in number during cancer recurrence and therapy resistance [112, 113]. Another study on breast cancer demonstrates that depletion of CD44 substantially hampers the aggregation of circulating tumor cells (CTCs) which contribute to cell migration, accompanied by down-regulation of p21-activated kinase 2 (PAK2) [30]. Via its N-terminal region, the intercellular interaction between CD44 and CD44 homophilic drives multicellular aggregation, and then triggers the interaction between CD44 and PAK2 to further activate focal adhesion kinase (FAK) pathway [30]. Single tumor cell aggregated, which enabled cluster formation and colonization of clustered tumor cells and contributed to tumor metastasis and secondary tumor formation in TNBC [30]. In mesenchymal TNBC which is a subcategory of TNBC and is featured with high rates of invasion and metastasis according to Lehmann BD’ work [57, 114], stable deletion of CD44 with shRNA vectors restrained proliferation, colony formation and invasion of SUM159, MDA-MB-436 and MDA-MB-231 [115].

According to immunohistochemistry analysis of colorectal cancer tissues, CD44 protein abundance was directly substantially associated with tumor grading, peritumoral budding, lymph node metastasis as well as advanced cancer stage [35]. The work by Wang et al. suggested that CD44 knockdown drastically restricted cancer motility and invasion of colorectal cancer (CoCa) cells due to distorted cooperation with associated integrins and reduced protease expression, respectively [116]. In colon cancer HCT116 cells, knockdown of CD44 with small interference RNA substantially hampered cell proliferation, migration and invasion and enhanced cell apoptosis, accompanied by suppression of the phosphorylation of Akt and glycogen synthase kinase-3β (GSK-3β), a decrease in the expression of Bcl-2 and Bcl-xl, and upregulation of caspase-3 and caspase-9 [117]. CD44v isoforms also participates in colorectal cancer progression. For instance, CD44v6 promotes the colonization, invasion and metastasis of colorectal CSCs [118]. In addition, CD44v6 expression in the medullary invasion front of mandibular-invasive OSCC was significantly higher in the group with cervical lymph node metastasis, in comparison to tumor tissues without lymph node metastasis [119], which suggested that CD44v6 facilitated lymph node metastasis of this tumor type.

Similar phenomena have also been observed in other tumor types, including glioblastoma [111], HNSCC [23], osteosarcoma [48], ovarian cancer [120], ESCC [100], and gastric cancer [121]. In human glioblastoma A172 and U251 cells, sevoflurane substantially enhanced the ability of migration and colony-forming in an concentration-dependent manner, but CD44 knockdown can reverse these effects, which indicates that CD44 is of great importance for sevoflurane-induced migration and colony-forming of tumor cells [111]. Angiogenesis is of great importance during tumor progression process [23]. In HNSCC, CD44 expression has been reported to be positively correlated with diverse pro-angiogenic genes based on the statistical analysis of the Cancer Genome Atlas data, and CD44 is enriched in human HNSCC tissues and is positively relevant with blood vessels based on immunohistochemistry analysis of tissue microarrays [23]. In addition, CD44-positive cells displayed remarkably higher microvascular density and expressed pro-angiogenic factors in comparison with CD44-negative cells or unsorted subgroup [23]. In ESCC, tumor tissues at the invasive front of tumors and metastatic lymph nodes expressed higher CD44v9, in comparison with cancer tissues at the center of tumors and primary tumors, respectively [100]. CD44v9 also contributed to inflammation-associated cancer development. Higher expression of CD44v9 was detected in *Opisthorchis viverrini*-related cholangiocarcinoma tissues than non-*Opisthorchis viverrini*-related cholangiocarcinoma [122]. In another study, metabolome analysis of 110 metabolites in CD44v9-positive and CD44v9-negative tumors suggested that CD44v9 could increase pentose phosphate pathway flux and sustain glutathione expression in gastric cancer cells [121].

Apart from the promoting effects CD44 exerts on cancer progression, aberrant expression of CD44 can also contribute to therapy resistance during anti-tumor management. Compared with CD44 negative human colorectal cancer cells (COLO 201), CD44 positive cells showed stemness characteristics and displayed lower sensitivity to the anti-tumor drug 5-FU, accompanied by the up-regulation of tumor stemness and chemoresistance-associated genes [107]. According to Zhang J’s work, high percentage of cervical cancer cells with the expression of CD44 and CD24 showed resistance to radiation treatment with expression profile of EMT [123]. In gastric cancer, upregulation of intracellular glutathione and suppression of 5-FU-induced accumulation of reactive oxygen species lies behind that CD44v9 contributed to resistance to 5-FU [124]. In OSCC, cells derived from both tumors and tumor margins expressed CD44, had the ability to form spheroids and displayed chemoresistance [125]. As observed, there was a significant rise in CD44s expression after high-dose X-ray exposure, which promoted longer-term cell survival after the irradiation via preserving Erk phosphorylation and radiation-induced EMT [126].

### CD44 and prognosis of cancer patients

CD44 can be a promising candidate for predicting the prognosis of cancer patients [85, 127–132]. Based on immunohistochemistry analysis of 125 breast cancer patient samples, it was found that CD44 protein level was positively correlated with poor disease-free survival (DFS) and OS [133]. According to immunohistochemistry analysis of a total of 206 RCC samples, high protein abundance of CD44 was correlated with malignant phenotype and unfavorable clinical outcomes of clear cell RCC rather than another two RCC subtypes (papillary and chromophobe RCC) [41]. Corresponding to the meta-analysis enrolling a total of 583 pancreatic cancer patients, high level of CD44 was correlated with adverse 5-year OS and advanced TNM stage, whereas did not associate with tumor size and tumor differentiation [42]. High expression of CD44 alone predicts poor overall survival, and simultaneous expression of CD44 and Aldehyde dehydrogenase 1 is linked to extremely unfavorable overall survival among endometrial cancer patients [130].

According to the results of a meta-analysis based on fifteen studies enrolling a total of 1633 tumor patients, positive expression of CD44v9 was associated with poor overall survival and relapse-free survival, compared with cancer patients with negative expression of CD44v9 [127]. In ESCC, higher protein level of CD44v9 at the invasive front of tumors was substantially correlated with worse OS and recurrence-free survival (RFS) [100]. According to both univariate and multivariate survival analysis, CD44v9 is an adverse independent prognosis predictor for five-year RFS among gastric cancer population [121]. High CD44 serum concentration and CD44v6 expression are remarkably associated with local recurrence and adverse clinical outcomes in oral cancer [128]. Besides, the mRNA expression of CD44v8-10/CD44s remarkably augments according to age of patients which is the well-established prognostic factor of papillary thyroid carcinoma [22].

However, some other studies have shown contrary or contradictory results for the role of CD44 in tumor-related clinical outcomes, such as breast cancer [134] and glioblastoma [135]. A meta-analysis of enrolling a total of 1747 breast cancer cases displayed that there was no any association between CD44 expression and OS [134]. As reported, CD44 could not serve as prognosis predictor of ovarian cancer [136]. CD44 has been demonstrated to have no effects on the OS and disease-free interval of epithelial ovarian cancer patients [136].

### CD44 and the development of anti-tumor drugs

During the past decades, enormous efforts have been exerted to develop novel effective anti-tumor drugs. Although much advancement has been achieved in cancer treatment, there is still a tumor population reacts poorly to current anti-tumor drugs. Recently, aiming at killing CSCs has become a promising therapeutic strategy for cancer population [137], and substantial interest has been emerged in the exploration of specific therapies targeting stemness-related marker of CSCs. CD44, as a well-known constituent of CSC niche, is among the chief potential rewarding anti-cancer targets for tumor management.

According to Moon HJ’s work, nonsteroidal anti-inflammatory drugs (NSAIDs) could reverse the resistance of human chronic myeloid leukemia K562 cells with high CD44 expression to 17-AAG (a Hsp90 inhibitor) and sensitize these cells to 17-AAG, which indicates that NSAIDs in coordination with Hsp90 inhibitor might synergistically potentiate the eradication effects on CSCs with CD44-overexpression [138]. Versini A, et al. evaluated the biological activity of salinomycin derivatives in both transformed human mammary epithelial cells with CD24^low^/CD44^high^ or CD24^high^/CD44^low^, the results of which revealed that the structural alternation derivative 4 showed a remarkably low half maximal inhibitory concentration value (IC50) against CD24^low^/CD44^high^ cells [137].

Antibodies to CD44 are being investigated for cancer therapy [139]. For instance, monoclonal antibody (mAb) U36 specific to CD44v6 showed remarkably high uptake in HNSCC [140]. Another mAb (VFF18) to CD44v6 derived from murine displayed fast and selective tumor uptake in human squamous cell carcinomas [141]. Besides, another study showed that a humanized mAb for CD44 (RG7356) exerted cytotoxic effects to leukemia B cells but had no effects on the viability of normal B cells in chronic lymphocytic leukemia [142]. CD44 mAb developed by Roche has been tested in several clinical trials for evaluating pharmacokinetics, pharmacodynamics, safety, and efficacy of this mAb among patients with advanced tumors harboring CD44 expression. As compared to Protein and Peptide-Based Approaches, cell-based panning represents the most efficient approach for isolation of a specific single domain antibody fragment to CD44 with more specificity based on a synthetic phage displayed library [143].

Apart from CD44 itself as a promising target in cancer treatment, it has also been suggested to probably act as a biomarker for anti-tumor drug targeting to CD44-positive cancer cells [144, 145]. The ubiquitin-specific protease USP22 is recognized to drive cancer invasion and metastasis as well as maintain CSCs [144]. Yang F’s work showed that the nanoliposomes composed of USP22 siRNA and CD44 antibodies conveyed USP22 siRNA to CD44-positive gastric CSCs and enhanced the therapeutic effects in comparison with nanoliposomes lack of CD44 antibodies [144]. In a recent research by Zhang M, et al. a programmable drug delivery system which is composed of a chondroitin sulfate hydrogel shell and hydrophobic cores was built for delivering anti-tumor drugs into drug-resistant cancer cells and keeping effective drug concentration to sensitizing cancer cells to anti-cancer drugs by down-regulation of the anti-apoptosis protein Bcl-xl [146]. This delivery nanoparticle could target tumor-specific CD44 molecule [146].

CD44 can be a target for HA-coated anti-tumor liposomes towards CSCs [147]. For instance, HA-coated cationic liposomes containing cabazitaxel (a tumor cell inhibitor) and silibinin (a CSC inhibitor), displayed enhanced cytotoxicity with low IC50, hampered cell migration, and triggered apoptosis among human prostate tumor cells with CD44 expression [147]. HA-coated nanoparticles containing anti-tumor drugs could also target CD44-positive cancer cells with high specialization and efficient drug delivery, refining the current anti-cancer management [148–153]. It has been observed that a rationally designed nanosystem containing gold nanostar/siRNA of heat shock protein 72/HA is endowed with the property of selectively sensitizing CD44-positive TNBC cells to hyperthermia, and improves the therapeutic accuracy and efficacy to TNBC with decreased unpleasant side effects both in vitro and in vivo [153]. As has also been displayed in Alamgeer M’s work, CD44s-positive small cell lung cancer cells benefit more from hyaluronic acid-irinotecan-carboplatin treatment [154].

As CD44 acts as the receptor for HA which can drive cancer migration, expansion, and metastasis [155], blocking HA-CD44 interaction by the degradation of HA or competitive suppression of CD44 might be also a promising strategy for tumor management. However, high spending and lack of specificity challenges [155]. For example, hyaluronidase which can accomplish the degradation of hyaluronic acid is difficult to be purified at the industrial level [155].

## Conclusions

**Fig. 3:**
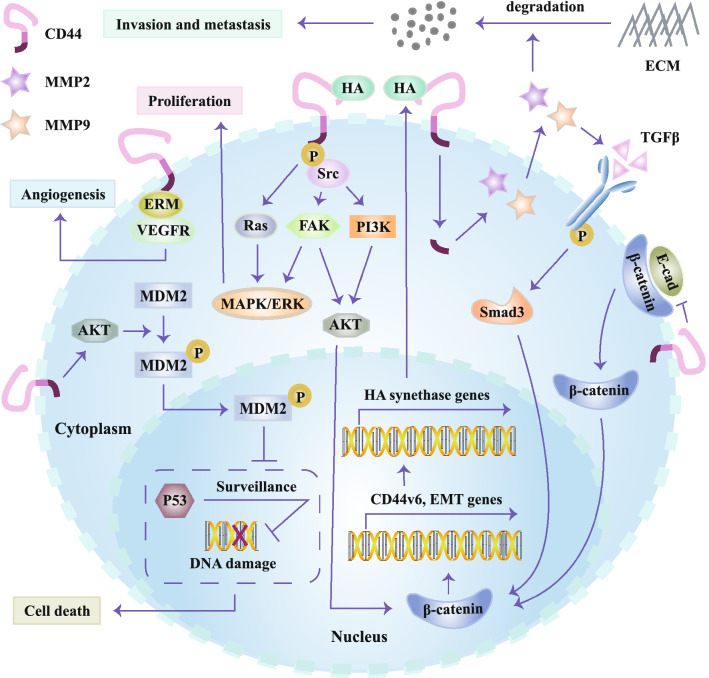
Signaling pathways which CD44 regulates. CD44v6 can enroll ERM proteins which can interact with VEGFR, contributing angiogenesis. CD44 activates AKT to promote the phosphorylation and nuclear translocation of MDM2, which blocks p53 genomic surveillance response. Subsequently, damaged hepatocytes escape from p53-mediated death, and carcinogen-induced mutations are maintained and transferred from parental cells to daughter cells, ultimately contributing to tumorigenesis. The combination of HA and CD44v6 promotes the phosphorylation of intracellular domain of CD44v6, which then activates Ras and FAK via Src and activates MAPK/ERK signaling. CD44v6 in combination with HA also promotes the PI3K/Akt signaling pathway and increases apoptosis. The intracellular tail of CD44v6 increases MMP2 and MMP9, which degrades ECM and promotes maturation of TGFβ. After binding to its receptor, TGFβ stabilizes β-catenin intracellularly via Smad3 together with activated Akt. Nucleus β-catenin stabilization enhances the expression of EMT-related genes and the gene encoding CD44v6. CD44v6 up-regulation enhances the expression of HA synthase genes, which promotes HA production. CD44 contributes to the dissociation of E-cadherin and β-catenin through suppression of E-cadherin, and then β-catenin translocates to nucleus

A growing body of evidence has demonstrated that CD44 is aberrantly up-regulated among diverse tumors in the forms of CD44s or CD44v. Herein, we aim at encapsulating the current understanding for CD44 structure and roles of CD44 during cancer initiation and progression. CD44 participates in the regulation of multiple signaling and pathways (Fig. 3), and in turn its expression is also regulated by a variety of molecules, such as transcription factors, microRNAs as well as post-translational modifications. CD44 exerts its effects on tumors mainly through stimulating signaling pathways that play vital roles in proliferation, apoptosis, EMT process and drug-resistance as well as activating transcription factors. However, the roles of diverse CD44 isoforms on cancer initiation and progression remain lack of further extensive investigation. Numerous studies demonstrate CD44 to be a potential therapeutic target among various cancers (Table 1). Potential therapeutic strategies targeting CD44-positive tumors via effectively blocking CD44, destroying HA-CD44 balance and increasing cellular concentration of anti-tumor drugs generate hope for anti-tumor drug development.

**Table 1:** CD44-targeted therapy in some preclinical and clinical studies

CD44-targeted agents	Cancer type	CD44 function	Ref.
CD44 antibodies			
CD44 blocking antibody	Lung cancer	Suppressing the proliferation of A549 cells	[139]
Monoclonal antibody (mAb) U36 specific to CD44v6	Head and neck squamous cell carcinoma	High tumor uptake	[140]
mAb (VFF18) to CD44v6	Human squamous cell carcinomas	Fast tumor uptake	[141]
A humanized mAb for CD44 (RG7356)	Leukemia B cells	Cytotoxic effects	[142]
Anti-CD44 mAb (H4C4)	Melanoma	Blocking cell aggregation and aggregate coalescence	[108]
CD44-targeting therapy via hyaluronic acid (HA)			
USP22 small interfering RNA-loaded nanoliposomes with CD44 antibodies	Gastric cancer	Targeting CD44( +) gastric cancer stem cells	[144]
HA coated cationic liposomes of cabazitaxel (CBX) and silibinin (SIL)	Prostate cancer	Showing proficient cytotoxicity against CD44( +) cells	[147]
HA-coated gold nanorods conjugated with pH-sensitive groups and loaded with doxorubicin	Cancer	Enhancing the killing of cancer cells and the inhibition of tumor growth	[145]
Polyethylene glycol-HA nanoparticles conjugated with mitoxantrone	Breast cancer	Delivering toward CD44 receptor-positive MDA-MB-231 cells rather than the CD44-negative MCF-7 cells	[148]
Nanosystem containing gold nanostar/siRNA of heat shock protein 72/HA	Triple negative breast cancer	Selectively sensitizing CD44-positive TNBC cells to hyperthermia	[153]
A phase IIa study: HA-irinotecan and carboplatin versus standard irinotecan and carboplatin	Extensive-stage small cell lung cancer	Selectively delivering anti-tumor drugs to CD44-positive tumor cells with enhanced efficacy	[154]

## Data Availability

Not applicable.

## Abbreviations

Akt: Protein kinase B
Bcl-xl: B-cell lymphoma-extra large
TGF-β1: CD44: cluster of differentiation 44
CD44s: CD44 standard isoform
CD44v: CD44 variant isoforms
CSCs: Cancer stem cells
CTCs: Circulating tumor cells
DFS: Disease-free survival
ECM: Extracellular matrix
EMT: Epithelial-mesenchymal transition
Erk: Extracellular signal-regulated kinase
ERM: Ezrin/radixin/moesin
ESCC: Esophageal squamous cell carcinoma
FAK: Focal adhesion kinase
GAB2: Growth factor receptor bound 2-associated binding protein 2
GSK-3β: Glycogen synthase kinase-3β
HA: Hyaluronic acid
HNSCC: Head and neck squamous cell carcinoma
IC50: Half maximal inhibitory concentration value
ICD: Intracellular domain
IL: Interleukin
KHDRBS3: KH RNA binding domain containing, signal transduction associated 3
mAb: Monoclonal antibody
MAPK: Mitogen-activated protein kinases
MEK: Mitogen-activated protein kinase
MMP9: Matrix metalloproteinase 9
NFκB: Nuclear factor-kappa B
NNMT: Nicotinamide N-methyltransferase
NSAIDs: Nonsteroidal anti-inflammatory drugs
OS: Overall survival
OSCC: Oral squamous cell carcinoma
PAK2: P21-activated kinase 2
PDK1: 3-Phosphoinositide-dependent kinase 1
PFKFB4: 6-Phosphofructo-2-kinase/fructose-2,6-bisphosphatase 4
PFS: Poor progression-free survival
PI3K: Phosphoinositide 3-kinase
RCC: Renal cell carcinoma;
RFS: Recurrence-free survival
STAT3: Signal transducer and activator of transcription 3
TGF-β1: Transforming growth factor-β1
TNBC: Triple-negative breast cancer
VEGFR: Vascular endothelial growth factor receptor
5‐FU: 5-Fluorouracil
